# Impact of Short-Term Fluoride Supplementation in Pregnancy on the Maternal Microbiome, Pregnancy, and Neonatal Outcomes: A Pilot Randomized Controlled Trial

**DOI:** 10.7759/cureus.116487

**Published:** 2026-09-18

**Authors:** Anna M Powell, Kim Jones-Beatty, Laura M Ensign, Kevin M Delong, Irina Burd

**Affiliations:** 1 Department of Obstetrics and Gynecology, Johns Hopkins University School of Medicine, Baltimore, USA; 2 Department of Obstetrics and Gynecology, University of Maryland Medical Center, Baltimore, USA; 3 The Center for Nanomedicine, Johns Hopkins University School of Medicine, Baltimore, USA

**Keywords:** fluoride supplementation, oral microbiome, pregnancy surveillance, preterm labour, vaginal microbiome

## Abstract

Introduction

This report presents secondary, exploratory analyses of a double-blind randomized trial originally designed to determine whether fluoride supplementation during pregnancy reduced preterm birth (PTB). The trial was terminated early because of enrollment difficulties and was not powered for its original clinical endpoint. We therefore examined whether randomized fluoride supplementation was associated with oral or vaginal microbiome composition and descriptively evaluated gestational and neonatal outcomes.

Methodology

In the original double-blind randomized controlled trial, pregnant participants at 10-20 weeks of gestation were assigned to a daily capsule containing 3 mg fluoride or an otherwise comparable prenatal vitamin. Of 319 screened individuals, 47 were eligible, and 30 participants completed follow-up through delivery. Maternal urine, oral swabs, and vaginal swabs were collected during the second and third trimesters and at delivery. The V3-V4 region of the bacterial 16S rRNA gene was sequenced from oral and vaginal specimens. The present secondary analyses compared microbiome composition and diversity by treatment assignment and descriptively examined gestational and neonatal outcomes.

Results

Urine fluoride concentration differed between groups at the second-trimester assessment but not at later assessments. No differences in vaginal microbiome richness or diversity were observed between treatment groups. Several oral taxa, including *Actinomyces*, *Actinomycetaceae*, and *Rothia mucilaginosa*, had nominally different abundances by treatment assignment; however, these exploratory comparisons were not adjusted for multiple testing. Overall oral alpha- and beta-diversity did not consistently differ by treatment group or visit. Eight positive correlations were observed between oral and vaginal taxa. Clinical outcomes did not show a consistent pattern by treatment assignment, and the study was not powered to assess maternal or neonatal efficacy or safety.

Conclusions

In this early-terminated pilot trial, prenatal fluoride supplementation was not associated with detectable differences in vaginal microbiome diversity and was not consistently associated with oral microbiome diversity. Nominal taxonomic and clinical differences should be considered hypothesis-generating because of the small sample size and multiple unadjusted comparisons. These data do not establish clinical benefit or harm from fluoride supplementation during pregnancy.

## Introduction

A growing body of evidence links dysregulated inflammatory responses to a range of adverse pregnancy outcomes. Activation of the inflammasome within gestational tissues is strongly associated with spontaneous preterm labor and chorioamnionitis. Chronic low-grade inflammation also impairs insulin signaling, promoting gestational diabetes, while placental inflammatory infiltrates disrupt nutrient transfer in fetuses with intrauterine growth restriction [1].

Preterm birth (PTB), or live birth prior to 37 weeks, is one of the leading causes of neonatal morbidity and mortality worldwide [2]. Overwhelming evidence implicates infection and inflammation in a majority of spontaneous PTBs [3,4]. Human host-microbiome interactions may act as drivers of infection and inflammation within the maternal-fetal interface and reproductive tract. Deep sequencing of maternal microbiomes allows for expanding awareness of host-microbiome interactions and more comprehensive investigations into the composition of microbiomes in the context of normal pregnancies or adverse pregnancy outcomes [5]. Over the course of pregnancy, the vaginal microbiome converges on *Lactobacillus* dominance [6]. Vaginal microbiome dysbiosis in early pregnancy has been implicated in increased risk for PTB [7,8]. The oral microbiome is thought to remain stable in composition during pregnancy under normal conditions, though pregnant women experiencing PTB may have alterations, including higher abundance of *Porphyromonas gingivalis* [9,10].

Maternal periodontal disease appears to increase the risk for PTB and other adverse pregnancy outcomes [11-13]. Fluoride supplementation of municipal water was introduced to reduce dental caries and improve dental health [14]. However, there is a dearth of data on the impact of fluoride supplementation on the maternal microbiome, natural history of pregnancy, or neonatal outcomes.

In mouse models, low-dose fluoride supplementation showed a protective effect against infectious challenge via intrauterine lipopolysaccharide (LPS) injection [15]. The effect of fluoride supplementation on human pregnancy outcomes, including the vaginal microbiome, is unknown. Maternal fluoride supplementation in pregnant women has been studied with the outcome of preventing dental caries in their children [16,17] but without reports of pregnancy outcomes. Although controversial, fluoride has been hypothesized to passively diffuse across the placenta from mother to fetus when fluoride intake is low and stop when fluoride intake exceeds a certain threshold [18]. The impact of fluoride intake on the vaginal microbiome is not known. In rodent models, acute fluoride toxicity causes intestinal damage and microbial dysbiosis, though the effect of typical daily fluoride intake on human microbiomes is largely uncharacterized [19].

The original trial was designed to test whether fluoride supplementation beginning at 10-20 weeks of gestation reduced PTB. Because enrollment difficulties led to early termination, the trial did not reach its planned sample size and could not evaluate that clinical efficacy endpoint. The present report therefore focuses on secondary, exploratory comparisons of oral and vaginal microbiome composition by randomized assignment and descriptive assessment of gestational and neonatal outcomes. We hypothesized that fluoride exposure might alter oral microbial composition; analyses of vaginal microbiome features and clinical outcomes were exploratory.

## Materials and methods

Study design

Study Population

For this pilot double-blind randomized controlled trial, a total of 319 patients were screened. Forty-seven maternal-fetal dyads were eligible, 30 of which were enrolled through delivery. Maternal and fetal outcomes were compared following 3 mg fluoride supplementation (treatment) versus a prenatal vitamin (placebo). IRB approval was obtained through the Johns Hopkins IRB (protocol IRB00038838, approved in 2014). This trial was registered with ClinicalTrials.gov (NCT02536352), and reporting was in concordance with CONSORT guidelines [19,20]. All patients and members of the research team were blinded to the treatment arm.

The Good Manufacturing Practice facility at the University of Maryland School of Pharmacy supplied both intervention arms of the study. Johns Hopkins Hospital Pharmacy (JHH Pharmacy) randomized the participants and distributed the capsules. Pregnant women participating in this study were randomly assigned by JHH Pharmacy to either a treatment or control group during the prenatal phase. The participants and all research team members participating in the study were blinded. The interventional model was parallel assignment. Masking was quadruple (participants, care providers, investigators, and outcome assessors).

Inclusion criteria included recruitment of adult pregnant women (18-55 years of age) presenting between 10 and 20 weeks of gestational age at the time of recruitment who planned delivery at Johns Hopkins Hospital or Johns Hopkins Bayview Medical Center. Patients were excluded for use of prescription-strength, high-dose fluoride products (greater than 0.76% fluoride) prescribed by a dental professional (toothpastes, mouthwashes, topical treatments) and occupational exposure to fluoride. All over-the-counter toothpaste and mouthwash products were acceptable to use. Any participants consuming amounts of fluoride close to 10 mg/day were also excluded.

Study removal occurred if patients withdrew consent, demonstrated an elevated urine fluoride level above 3 mg/L, or needed to use high-dose fluoride products during the study as instructed by a dentist or other healthcare provider. In this case, participants were instructed to return the bottle of unused vitamins to ensure there was no continued use.

Maternal urine samples, oral and vaginal swabs were collected during the second and third trimesters and at delivery. Buccal and gingival swabs were performed by study staff by inserting the swab into the participants' mouths and twisting the cotton tip along the inside of their cheeks, along the surface of the teeth, and along their upper and lower gum lines twice bilaterally. Vaginal swabs were self-collected by participants by inserting the swabs two to three inches into their vaginal cavities and twisting the swab three to four times. The swabs were then placed into sterile biospecimen bags and given to the research coordinators for processing. Swabs were stored at -80°C.

DNA extraction

DNA was extracted from vaginal swab samples as described previously [20]. Briefly, BD ESwab fluid was resuspended in lysozyme-containing lysis buffer. Following lysozyme treatment, DNA was extracted using the DNeasy® Blood and Tissue Kit (QIAGEN®, Hilden, Germany), including the addition of proteinase K for pretreatment of Gram-positive bacteria. DNA from saliva and buccal swab samples was extracted using the QIAamp DNA Microbiome Kit (QIAGEN®, Hilden, Germany) following the manufacturer's instructions. Briefly, human host DNA was depleted. Mechanical and chemical lysis were then performed to disrupt bacterial cells. DNA was eluted using QIAGEN columns provided in the kit, and the extracted DNA was quantified using a NanoDrop.

DNA amplification and library preparation

Vaginal swab library preparation was performed according to the Illumina 16S Metagenomic Library Preparation protocol (Illumina, San Diego, CA, USA) using primers that amplify the V3-V4 region of the 16S gene. Paired-end sequencing of the pooled library was performed using the MiSeq system (Illumina, San Diego, CA, USA) by the Genetic Research Core Facility (GRCF) (Johns Hopkins Medical Institute, Baltimore, MD, USA), generating 2 × 250-bp reads. Adapter and barcode trimming was also performed by the GRCF.

For oral swab library preparation, libraries for the V3-V4 region of the 16S rRNA gene were constructed in accordance with the 16S Metagenomic Sequencing Library Preparation protocol (Part #15044223 Rev. B; Illumina, San Diego, CA, USA).

Sequence processing

Vaginal Samples

QIIME 2 2020.2 was used to join paired reads and demultiplex samples in combination with DADA2 to trim and denoise samples. Taxonomic assignments were given to representative sequences in QIIME 2 using the pre-fitted, sklearn-based taxonomy function and a taxonomic classifier built from a previously described custom reference database [20] that combines an existing vaginal microbiome 16S database [21] with the V4 regions of additional species represented in the VaHMP V1-V3 database [7].

Oral Samples

DNA from saliva and buccal swab samples was extracted using the QIAamp DNA Microbiome Kit (QIAGEN, Hilden, Germany) following the manufacturer's instructions. Briefly, human host DNA was depleted. Afterwards, mechanical and chemical lysis were performed to disrupt bacterial cells. DNA was eluted using QIAGEN columns provided in the kit, and the extracted DNA was quantified by NanoDrop. Libraries for the V3-V4 region of the 16S rRNA gene were constructed in accordance with the 16S Metagenomic Sequencing Library Preparation protocol (Part #15044223, Rev. B; Illumina, San Diego, CA, USA).

Sequencing Data Analysis

Vaginal microbiome reads were rarefied in R (R version 3.4.3; R Foundation for Statistical Computing, Vienna, Austria), and partitioning around medoids (PAM) clustering was performed to assign samples to community state types (CSTs). Inverse Simpson indices were calculated in R using the vegan (version 2.5-4) diversity command and a Bray-Curtis dissimilarity matrix.

Oral sample sequencing produced between 120,000 and 1,331,000 paired-end reads per sample (average, 331,000 pairs; 662,000 reads), with an average read length of 250 bp. These reads were analyzed to: (i) determine metagenomic (Operational Taxonomic Unit (OTU)) profiles of each sample; (ii) calculate and compare intra- (alpha) and intergroup (beta) diversity; (iii) identify differentially represented taxa; and (iv) determine potential correlations with the previously analyzed vaginal microbiome from the same patients. We used the QIIME2 [22] and MetaStats 2.0 [23] software packages and custom R scripts. Reads were demultiplexed and filtered to remove low-quality data, vector contaminants, and chimeric reads. Following testing to determine optimal trimming, filtering, and mapping criteria, only read 1 sequences were retained for subsequent analyses, with reads trimmed to position 13 at the 5′ end and from position 160 at the 3′ end, resulting in 147-bp-long sequences (all other options tested, including using read 2 and all paired-end reads, had lower performance due to low sequence quality).

Read assignment to OTUs was performed using the QIIME2 function “feature-classifier” with the pre-fitted, sklearn-based taxonomy classifier “classify-sklearn” and a taxonomic classifier based on the GreenGenes database clustered at 99% sequence identity. Taxonomic frequency profiles were created to reflect the community’s OTU composition at different phylogenetic levels.

Rarefaction plots of within-group (alpha) diversity and between-group (beta) diversity were computed with the “diversity core-metrics-phylogenetic” function. Comparisons between groups of alpha diversity values (for “faith_pd,” “evenness,” “Shannon,” and “observed_features” vectors) were performed, and statistical significance was calculated using the “alpha-group-significance” function. To test whether the fluoride treatment and other categories or combinations of categories were associated with significant differences in the weighted and unweighted UniFrac distances, we used the “beta-group-significance” function. Lastly, the MetaStats 2.0 package was used to compare metagenomic profiles at each phylogenetic level (levels 1-7), from kingdom to species, to determine taxa that were statistically enriched or depleted in one condition compared with the other (alternatively, the ANCOM method in QIIME2 was used to perform differential abundance tests; however, the results were less interpretable).

Vaginal microbiome results were then classified as either individual bacterial OTUs or CSTs. The communities can be clustered into five groups: four were dominated by *Lactobacillus iners*, *Lactobacillus crispatus*, *Lactobacillus gasseri*, or *Lactobacillus jensenii*, whereas the fifth had lower proportions of lactic acid bacteria and higher proportions of strictly anaerobic organisms [24]. 

Heatmaps

Heatmaps were generated to display the abundance levels of taxa represented in the oral and vaginal microbiomes of the samples (patient × timepoint) to aid in visually identifying potential correlated taxa and/or groups of patients. On the heatmap, rows represent taxa (either oral or vaginal), and columns represent the “patient × timepoint” class. Both absolute-value and Z-score-normalized (per row) maps were generated, and clustering was performed to group rows, columns, both, or neither.

To curate data for inclusion in the heatmap, only patient × timepoint combinations for which both a vaginal and an oral sample were available were included (42 out of the original 47). Then, low-abundance and potentially artifactual taxa (<10 reads in both the “fluoride treatment” and “no treatment” sample groups) were excluded. Heatmaps were generated in R with the heatmap.2 function.

Correlations

To search for correlated taxa between the patients’ vaginal and oral microbiomes, we started by identifying the common taxa at the species level between the two datasets, which were analyzed with different tools and differed in their composition and location in the body. We further filtered out underrepresented and potentially artifactual taxa (abundance <10 reads). We then performed the following correlation analyses across patient × timepoint vectors using the rcorr function in R: (i) between the same taxon abundance in the oral and vaginal microbiomes, to verify whether levels or variations in the taxon’s abundance in the oral microbiome matched those in the vaginal microbiome; and (ii) between any filtered oral and vaginal taxa, to determine potential co-responding taxa in the two microbiomes.

Sample size calculation and data analysis

The original primary outcome was PTB after initiation of fluoride supplementation at 10-20 weeks of gestation. The planned trial required a substantially larger sample to evaluate this endpoint. Enrollment was halted after two years because of recruitment difficulties, and only 30 participants completed follow-up through delivery. Consequently, all microbiome and perinatal analyses in this report should be considered secondary, exploratory, and hypothesis-generating.

Statistical analysis

Statistical analyses were performed using SAS version 9.4 (SAS Institute, Cary, NC, USA) and Stata version 18 (StataCorp, College Station, TX, USA). Categorical variables were compared using chi-square or Fisher’s exact tests, as appropriate. Continuous variables were assessed for normality using the Shapiro-Wilk test and summarized as mean (standard deviation) or median (interquartile range), as appropriate. Between-group comparisons used Student’s t-tests or Wilcoxon rank-sum tests. Linear mixed-effects models were used for repeated measurements of urine fluoride concentration and microbiome diversity, with treatment group, visit, and urine fluoride concentration specified as fixed effects and participant as a random effect, as applicable. Taxon-level comparisons used nonparametric tests because relative abundances were non-normally distributed. Because this was an exploratory pilot analysis, taxon-level and clinical comparisons were not adjusted for multiple testing; p-values are nominal and should be interpreted as hypothesis-generating rather than confirmatory.

Vaginal microbiome measurements of richness (abundance) and alpha diversity (Inverse Simpson, Shannon, and CST assignment) and oral microbiome alpha diversity (Shannon and Faith’s phylogenetic diversity (PD)) were also modeled as response variables, with treatment group, visit time, and urine fluoride concentration as fixed effects, while subject identification was used as the random effect. To ensure sensitive detection of signals of interest, we primarily focused on changes in microbiome features per patient between baseline and the second trimester, from the second to the third trimester, and from baseline to the third trimester. The distribution of taxon-specific relative abundances was non-normal, and nonparametric Mann-Whitney test p-values are reported where appropriate.

Oral swabs were selected for analysis if participants also had vaginal swab data available. Oral microbiome read counts were normalized by sample size (to match the median sample). Low-abundance taxa (i.e., with ≤15 reads on average across both the "fluoride" and the "no treatment" conditions) were cleaned up. Next, heatmaps were generated using (i) the (normalized) read counts of taxa and (ii) the Z-score-normalized read counts (i.e., the average abundance for the taxon in all samples is computed, and sample counts are Z-score transformed, $x \rightarrow \frac{(x-\mathrm{avg})^2}{\mathrm{avg}}$. Values were plotted in read counts and in log-transformed ("lg" files), and clustering was performed three ways: (i) by rows only (to find correlated taxa); (ii) by rows and by columns, to identify correlated taxa as well as (patient, time) groups that exhibit these patterns; and (iii) by columns only (to identify (patient, time) groups that have similar responses to treatment or no treatment).

To analyze overlap between oral and vaginal microbiome samples, data from each OTU table were merged, and only samples present in both datasets were kept (42 of 48 in the oral dataset). Normalization was performed; only taxa with an average abundance per treatment >10 were retained, where the average abundance in the treatment or placebo groups was >10. Heatmaps were generated using absolute values (normalized counts). Absolute values were scaled and centered by rows using Z-score normalization. For each set, the matrix was prepared with and without log transformation.

## Results

A total of 319 patients were screened; 272 patients did not consent. Forty-seven maternal-fetal dyads were eligible, but four patients withdrew before randomization and 13 withdrew post-randomization. Thirty patients were enrolled through delivery. Sixteen patients were assigned to the placebo group, and 14 were randomly assigned to fluoride supplementation (Figure 1).

**Figure 1:**
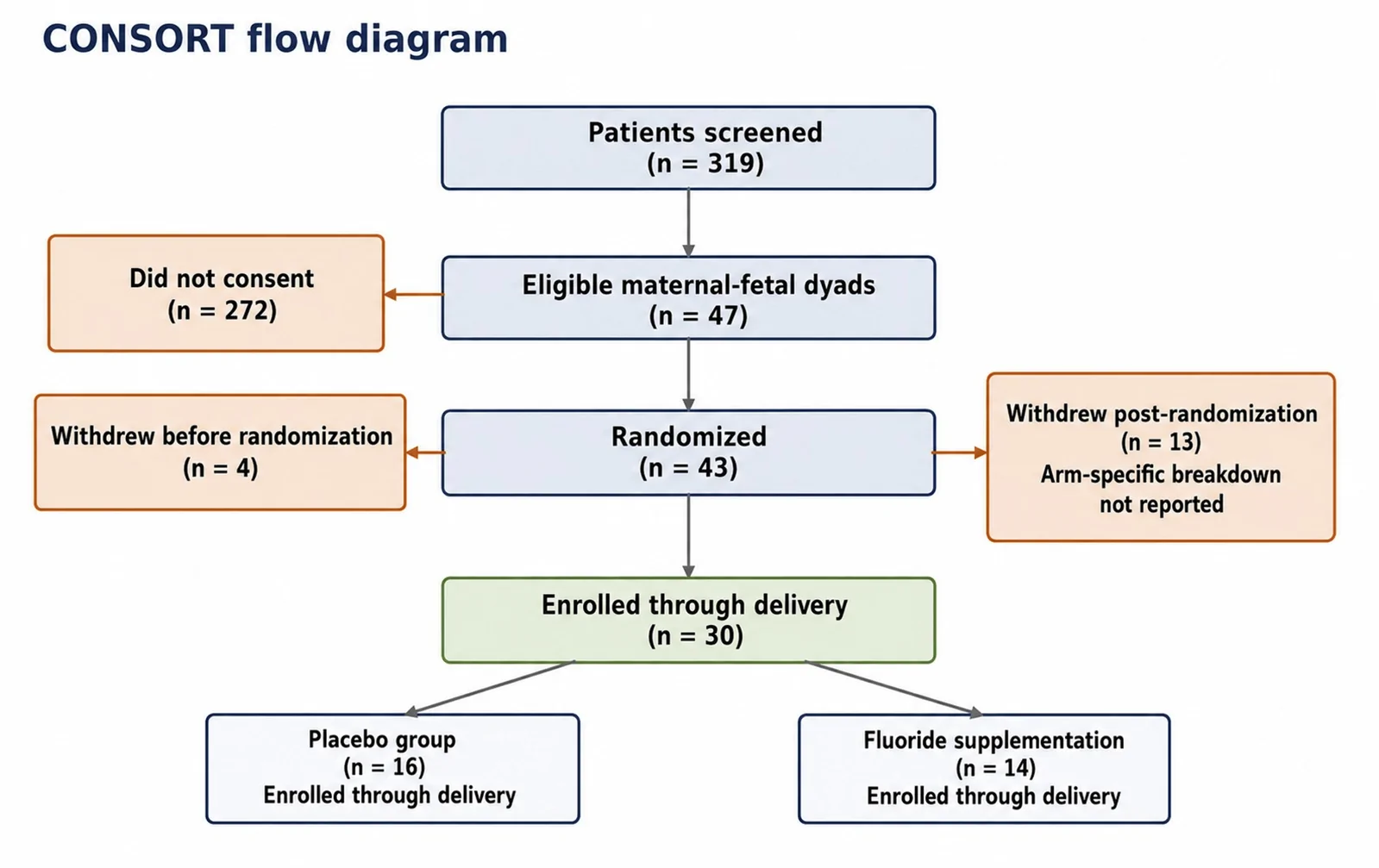
CONSORT Flow Diagram

The mean age of participants was 33.8 ± 3.9 years, and participants did not significantly differ in race/ethnicity by treatment group assignment (Table 1). The median gestational age at delivery was 38.6 (range, 37.1-39.4) weeks. Among the randomized participants, those receiving fluoride supplementation had higher second-trimester urine fluoride concentrations (median, 0.79 ng/mL (range, 0.46-0.97) vs. 0.6 ng/mL (range, 0.49-0.76); p = 0.03) but similar third-trimester and delivery urine fluoride concentrations. There were no differences in maternal mode of delivery (vaginal delivery or cesarean section) or PTBs (Table 1).

**Table 1:** Demographics, Maternal and Neonatal Outcomes of Routine Care Versus Fluoride Supplementation in Pregnancy

	Control group, N = 16	Fluoride supplementation, N = 14	p-value
Age	34.6 ± 2.8	33.8 ± 4.9	0.6
Race: African American	5/16 (31.3%)	6/14 (42.9%)	0.55
Race: Caucasian	10/16 (62.5%)	8/14 (57.1%)	0.55
Race: Asian	0/16 (0%)	0	0.55
Race: Other	1/16 (2.4%)	0	0.55
EGA delivery	38.6 (37.2-39.6)	38.7 (35-39.4)	0.55
Cesarean delivery	5/16 (31.3%)	5/13 (38.5%)	0.68
Neonatal birthweight (g)	3069 ± 672	3254 ± 546	0.05
Neonatal delivery length (cm)	49.8 (48.3-51.8)	50.4 (48.5-51)	0.97
Neonatal delivery head circumference	33 (32-35)	34 (32-35)	0.54
Female neonate	5/14 (35.7%)	7/13 (53.9%)	0.34
Male neonate	9/14 (64.3%)	6/13 (46.5%)	0.34
Apgar 1 minute	8 (7-8)	8 (8-8)	0.11
Apgar 5 minutes	9 (9-9)	9 (9-9)	0.05
Multiple gestation (yes)	2/16 (12.5%)	0/14	0.3
Neonatal anomaly	2/14 (14.3%)	2/13 (15.4%)	0.4
Neonatal antibiotics within 1st week of life	5/16 (31.3%)	4/14 (28.6%)	0.31
Neonatal fever	0/14	2/12 (16.7%)	0.2
Neonatal comorbidity	7/16 (43.8%)	8/14 (57.1%)	0.46
Neonatal illness during hospitalization	0/14	1/11 (9.1%)	0.11
NICU admission	2/16 (12.5%)	3/14 (21.4%)	0.31
Neonatal weight at discharge (g)	3055 (2878-3295)	3418 (2750-3538)	0.3
Median Maternal fluoride measurements (range) (ng/mL)			
2nd trimester (urine) (visit 1-baseline)	0.52 (0.44-0.76)	0.79 (0.57-0.94)	0.02
3rd trimester (urine) (visit 2)	0.94 (0.63-1.8)	1.1 (1-1.8)	0.21
Delivery (urine) (visit 3)	0.45 (0.39-0.64)	0.41 (0.24-0.78)	1.0

Neonatal outcomes did not show a consistent pattern by treatment assignment. Although nominal between-group differences were observed for selected Apgar and fever comparisons in some analyses, the small number of participants and events and the absence of adjustment for multiple testing preclude conclusions regarding neonatal benefit or harm. Birth weight, length, head circumference, neonatal intensive care unit admission, congenital anomalies, comorbidities, and illness during hospitalization were otherwise similar between groups (Table 1).

Vaginal microbiome

Seventy-six microbiome samples were collected from the 42 randomized participants and analyzed; 71 passed sequencing and quality-control criteria (35 from the placebo group, 26 from the fluoride supplementation group). While baseline CSTs differed by maternal race, there were no significant differences in vaginal microbiome richness or diversity between treatment groups at any time point (Table 2). 

**Table 2:** Vaginal and Oral Microbiome Parameters

Maternal vaginal microbiome parameters			
	Placebo (n = 35)	Fluoride supplementation (n = 26)	p-value
Read summary (total)	20033.11 ± 7517.66	19632.88 ± 5699.87	0.55
Read summary (T1, n = 14/12)	21583.64 ± 7647.30	20048.08 ± 5330.34	0.56
Read summary (T2, n = 15/10)	19141.53 ± 8206.44	20031.50 ± 6623.91	0.31
Read summary (T3, n = 6/4)	18644.17 ± 5679.16	17390.75 ± 5196.88	0.55
Inverse Simpson (total)	3.13 ± 4.14	4.29 ± 6.95	0.22
Inverse Simpson (T1, n = 13/12)	2.50 ± 2.00	3.09 ± 2.56	0.59
Inverse Simpson (T2, n = 15/10)	3.52 ± 5.34	2.83 ± 2.85	0.24
Inverse Simpson (T3, n = 5/4)	4.16 ± 4.74	12.55 ± 15.92	-
Shannon (total)	1.52 ± 1.07	1.27 ± 0.95	0.33
Shannon (T1, n = 13/12)	0.91 ± 0.80	1.27 ± 0.98	0.68
Shannon (T2, n = 15/10)	1.14 ± 0.95	1.16 ± 0.96	0.95
Shannon (T3, n = 5/4)	2.68 ± 0.95	1.67 ± 0.80	0.78
Maternal oral microbiome parameters			
	Placebo (n = 23)	Fluoride (n = 25)	p-value
Read Summary (total)	337748 ± 127456.3 (n = 23)	289226.7 ± 105225.8 (n = 25)	0.16
Read Summary (T1, n = 8/7)	307368.9 ± 96891.9	296435.6 ± 104522	0.84
Read Summary (T2, n = 10/11)	384003.9 ± 161493.6	291524.6 ± 129727.1	0.16
Read Summary (T3, n=5/7)	293842.8 ± 68075.3	278406.86 ± 71481.5	0.71
Faith PD (total)	14.74 ± 2.86	15.62 ± 3.85	0.19
Faith PD (T1, n = 8/7)	16.44 ± 3.63	14.24 ± 2.99	0.23
Faith PD (T2, n = 10/11)	14.07 ± 2.23	15.34 ± 2.95	0.28
Faith PD (T3, n = 5/7)	13.35 ± 1.23	17.45 ± 5.44	0.13
Shannon (total) T2 T3	20.91 ± 13.36	27.04 ± 12.75	0.06
Shannon (T1, n = 8/7)	3.56 ± 0.81	4.25 ± 0.73	0.11
Shannon (T2, n = 10/11)	3.82 ± 1.03	4.10 ± 1.05	0.55
Shannon (T3, n = 5/7)	3.94 ± 0.64	4.52 ± 0.87	0.21

Exploratory, unadjusted comparisons identified nominal changes in several vaginal taxa between visits. Because numerous taxa and intervals were evaluated without multiplicity correction, these findings are presented only as hypothesis-generating and should not be interpreted as evidence of a treatment effect. No species-level differences were observed between visits 1 and 3; Lachnospiraceae showed a nominal difference between visits 2 and 3 (p = 0.04). In exploratory regression models, PTB was not associated with vaginal Shannon diversity (adjusted odds ratio (aOR), 0.37; 95% CI, 0.03-4.98) or inverse Simpson diversity (aOR, 1.39; 95% CI, 0.51-3.78) after adjustment for study visit.

Heat maps representing microbiome comparisons between the vaginal placebo and fluoride groups are shown in Figure 2. Overall, minimal differences were noted among most commonly represented bacterial taxa.

**Figure 2:**
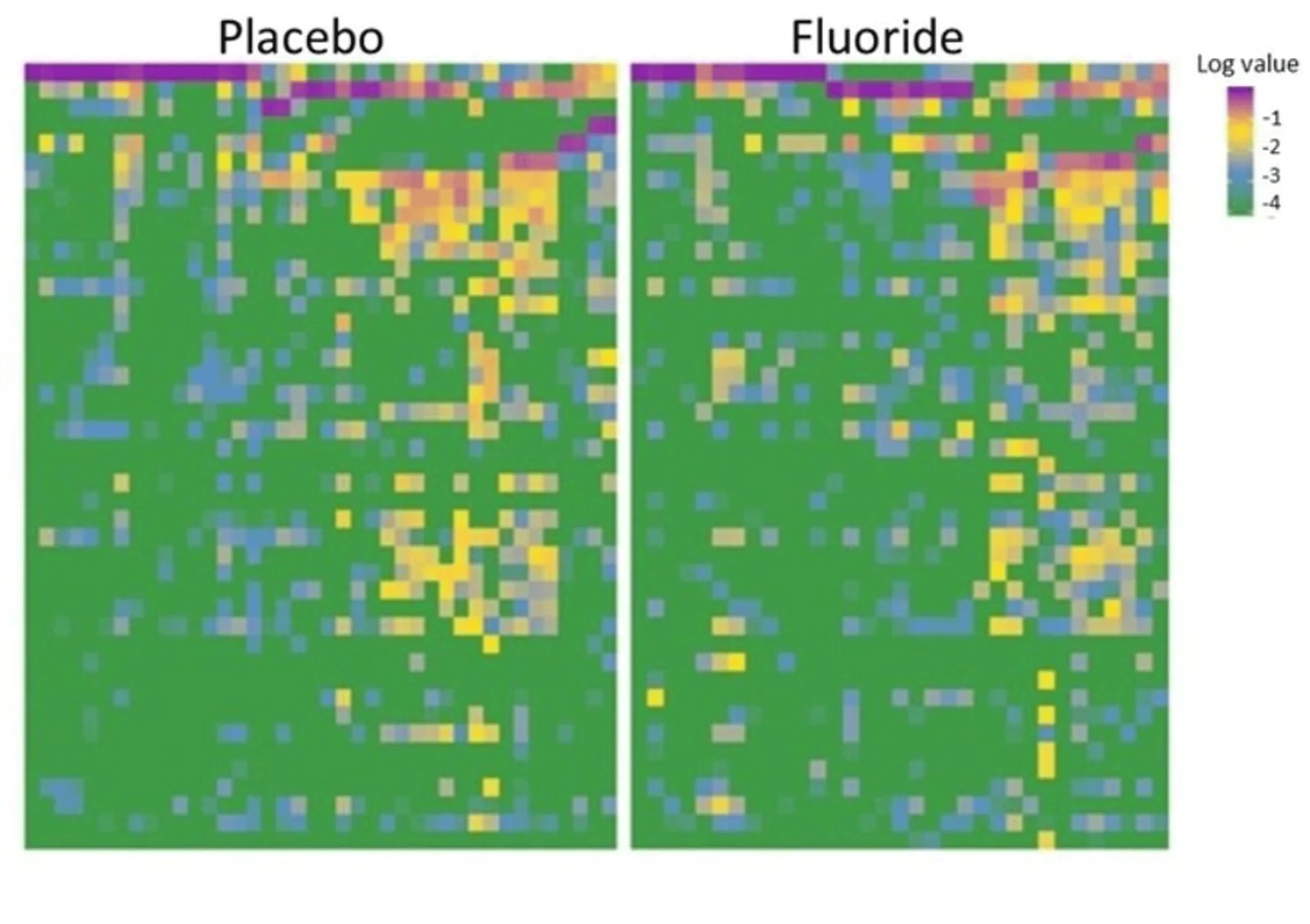
Vaginal Microbiome Taxon Heatmap by Treatment Group

Oral microbiome

Oral microbiome samples were collected from 25 participants (n = 49 samples total) in addition to vaginal swabs. Twenty-three samples were from the fluoride treatment group, and 25 samples were from the placebo group (Table 2). The top 10 most abundant oral taxa among treatment and control group samples included *Streptococcus* spp., *Streptococcus infantis*, *Veillonella* spp., *Veillonella dispar*, family *Gemellaceae*, *Rothia mucialaginosa*, *Rothia dentocariosa*, genus *Actinomyces*, *Selenomonas noxia*, and genus *Granulicatella*. Representative heat maps are shown in Figure 3. 

**Figure 3:**
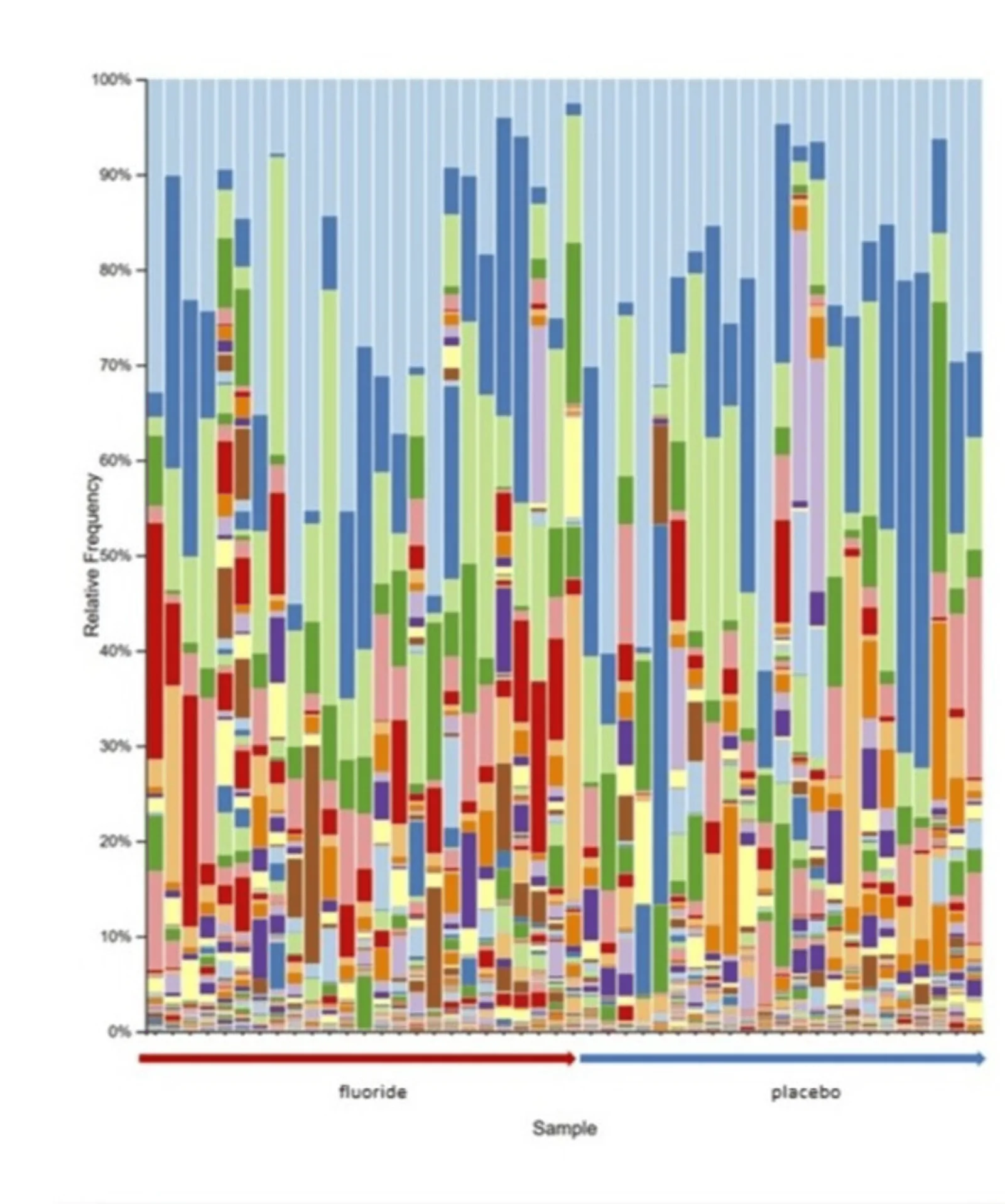
Oral Microbiome Taxon Heatmap By Treatment Group

Study participants in the fluoride supplementation group had higher overall read summaries of genus *Actinomyces* (p = 0.04), family *Actinomycetaceae* (p = 0.02), and *R. mucilaginosa* (p = 0.04). There were no significant differences in alpha diversity measurements overall by treatment group assignment: Faith’s PD (p = 0.37) and Shannon diversity (approaching significantly higher alpha diversity in the treatment group, p = 0.06), nor at individual study timepoints, as also demonstrated by nonsignificant differences in Pielou evenness. There were no significant differences in beta diversity by unweighted UniFrac between treatment groups (p = 0.412, data not shown) or by visit time (overall p = 0.48, data not shown), based on pairwise PERMANOVA calculations. Apart from three outlier values, there were no differences by treatment group over time based on ANCOM volcano plot W values (data not shown). Those experiencing PTB in the cohort (n = 4 with oral samples) had significantly lower Faith’s PD (PTB, 12.34 ± 1.87 vs TB, 15.5 ± 2.88; p = 0.03) but not Shannon diversity (PTB, 3.68 ± 0.37 vs TB, 4.03 ± 1.03; p = 0.29). In logistic regression modeling, PTB was also not significantly associated with oral Shannon diversity (aOR, 0.43; 95% CI, 0.06-3.25) or Faith’s PD (aOR, 2.58; 95% CI, 0.93-7.14) after adjusting for study timepoint. Shannon alpha diversity values were significantly associated with treatment group (p = 0.02) when controlling for visit time and urine fluoride concentrations, while Faith’s PD values were not associated with treatment group, visit time, or urine fluoride concentrations (data not shown).

Oral swabs were collected from 25 participants. Forty-nine specimens were collected, 48 passed sequencing and quality-control criteria (25 from the fluoride group and 23 from the placebo group), and 42 participant-time-point specimens had a corresponding vaginal sample. The most abundant oral taxa included *Streptococcus* spp., *Streptococcus infantis*, *Veillonella* spp., *Veillonella dispar*, *Gemellaceae*, *R. mucilaginosa*, *Rothia dentocariosa*, *Actinomyces*, *Selenomonas noxia*, and *Granulicatella*. Representative heat maps are shown in Figure 3.

In exploratory unadjusted analyses, *Actinomyces* (p = 0.04), *Actinomycetaceae* (p = 0.02), and *R. mucilaginosa* (p = 0.04) showed nominally higher abundance in the fluoride group. These findings did not remain part of a prespecified multiplicity-controlled analysis and should be interpreted cautiously. Overall Faith’s PD and Shannon diversity did not consistently differ by treatment group, and no differences in unweighted UniFrac beta diversity were observed by treatment group or visit. Participants with PTB who had oral samples available (n = 4) had lower Faith’s PD than participants with term birth but not lower Shannon diversity; these sparse exploratory comparisons cannot support inference about preterm-birth risk.

There were no significant differences in beta diversity by unweighted UniFrac between treatment groups (p = 0.412, data not shown) or by visit time (overall p = 0.48, data not shown), based on pairwise PERMANOVA calculations. Apart from three outlier values, there were no differences by treatment group over time based on ANCOM volcano plot W values (data not shown). Those experiencing PTB in the cohort (n = 4 with oral samples) had significantly lower Faith’s PD (PTB, 12.34 ± 1.87 vs TB, 15.5 ± 2.88; p = 0.03) but not Shannon diversity (PTB, 3.68 ± 0.37 vs TB, 4.03 ± 1.03; p = 0.29). In logistic regression modeling, PTB was also not significantly associated with oral Shannon diversity (aOR, 0.43; 95% CI, 0.06-3.25) or Faith’s PD (aOR, 2.58; 95% CI, 0.93-7.14) after adjusting for study timepoint. Shannon alpha diversity values were significantly associated with treatment group (p = 0.02) when controlling for visit time and urine fluoride concentrations, while Faith’s PD values were not associated with treatment group, visit time, or urine fluoride concentrations (data not shown).

Correlations between oral and vaginal microbiome

We then investigated correlations between vaginal and oral microbiomes in study participants with both samples available. Table 3 outlines bacterial genera with significant correlation coefficients between oral and vaginal taxa abundance.

**Table 3:** Correlations Between Oral and Vaginal Bacterial Taxa Taxa are listed in order of strongest positive to weakest positive correlation (top 2 = strongest positive correlations; bottom 3 = weakest positive correlations).

Oral	Vaginal	Correlation coefficient	p-value
Porphyromonas	Corynebacterium	0.68	0
Bacteroidales	Actinomyces	0.55	0
Clostridiales	Actinomyces	0.43	0
Lactobacillus	Streptococcus anginosus	0.45	0
Porphyromonas	Porphyromonas	0.44	0
Dialister	Actinomyces	0.41	0.01
Porphyromonas	Actinomyces	0.39	0.01
Porphyromonas	Dialister	0.33	0.03

## Discussion

Principal findings

In this pilot randomized controlled trial with premature study termination, we investigated the possible association between oral and vaginal microbiome changes over time and fluoride supplementation. To the best of our knowledge, this is the first comprehensive study addressing the effects of fluoride supplementation on maternal and fetal outcomes and their correlations with oral and vaginal maternal microbiome composition. Relevant reports on the topic do not address pregnancy or focus on maternal outcomes, respectively [18,25].

While fluoride supplementation does not appear to be associated with altered vaginal microbiome composition, richness, or alpha diversity, it did impact the oral microbiome, with enrichment of certain beneficial species such as *R. mucilaginosa*. We identified correlated vaginal and oral microbiome taxa based on abundance. Neonate data indicated significant improvements in Apgar scores. Overall, despite the small cohort size, the results suggest potential benefits of fluoride supplementation in pregnancy.​​​​

Clinical implications 

Fluoride has been widely used in public health to prevent dental caries, and water fluoridation was endorsed as a preventive measure. However, during pregnancy, maternal-fetal safety demands special consideration. As with many nutrients and environmental exposures, what is beneficial in one context may need reassessment when fetal neurodevelopment is involved. More recent studies [26] have raised questions about potential associations between higher maternal fluoride intake and subtle neurodevelopmental changes in children. These findings, while not yet definitive, highlight the need for further investigation. Given individual variability in fluoride exposure from water, toothpaste, and diet, blanket supplementation may not be warranted during pregnancy. A precautionary approach, involving individualized assessment and monitoring, is both safer and scientifically responsible. There is a critical need for updated clinical guidelines based on high-quality human data to ensure both maternal dental health and fetal safety. We advocate for expanded research and nuanced risk-benefit analysis specific to pregnancy.

Fluoride’s anti-cavity effect has been well studied but not entirely elucidated. Hypothesized mechanisms range from inhibition of microbial carbohydrate metabolism, reducing acid formation, disruption of intracellular pH regulation, inhibition of extracellular polysaccharide production, inhibition of enamel demineralization, and enhancement of remineralization [27]. An in vitro biofilm experiment exposed to sodium fluoride at different molar concentrations showed that fluoride did not affect bacterial colony numbers but prevented enamel mineral loss at 1400 and 400 ppm [27]. Fluoride has also demonstrated antibacterial activity by reducing acid production by saccharolytic bacteria through enolase inhibition in the glycolytic pathway. The full effects of fluoride on the composition of the oral microbiome are challenging to assess because fluoride use is so widespread [28]. While oral microbiome alterations have been linked to adverse pregnancy outcomes, there is no conclusive evidence that treatment of oral dysbiosis improves these outcomes. *R. mucilaginosa* is a commensal, facultative anaerobe considered a part of the normal oral microbiome as well as the respiratory tract; in non-pregnant individuals with chronic lung disease, its presence in the lower respiratory tract may mitigate inflammation, thus influencing the severity and progression of chronic respiratory disorders [29]. Oral *Actinomyces* are Gram-positive, facultative anaerobes considered normal oral microbiome residents, which may be implicated as initial colonizers of polymicrobial biofilms leading to oral cavities [30].

Strengths and limitations

Strengths of this study include the randomized and blinded study design intended to link the effects of fluoride supplementation in pregnancy on maternal and fetal outcomes and correlate these with the composition of maternal oral and vaginal microbiomes. A major limitation is that the study is underpowered to detect differences in treatment groups for the primary outcome of PTB. Interpretation of fluoride supplementation effects on the vaginal microbiome may also be limited by the relatively small sample size. Potentially, despite controlling for fluoride supplement exposure in prenatal vitamins, our study participants were inadvertently exposed to fluoride from additional sources, making it difficult to fully characterize its impact.

## Conclusions

Our study provides limited and preliminary evidence that there is a beneficial effect of fluoride supplementation on the oral microbiome in pregnancy. A larger, adequately powered cohort would be required to establish stronger associations with pregnancy outcomes.
